# Excessive Daytime Sleepiness Measurements in Children With Attention Deficit Hyperactivity Disorder

**DOI:** 10.3389/fpsyt.2020.00003

**Published:** 2020-02-26

**Authors:** Stéphanie Bioulac, Jacques Taillard, Pierre Philip, Patricia Sagaspe

**Affiliations:** ^1^ CHU Pellegrin, Clinique du Sommeil, Bordeaux, France; ^2^ Université de Bordeaux, Sommeil, Attention et Neuropsychiatrie, USR 3413, Bordeaux, France; ^3^ CNRS, SANPSY, USR 3413, Bordeaux, France

**Keywords:** attention deficit hyperactivity disorder (ADHD), Children, excessive daytime sleepiness (EDS), assessment, neuropsychological markers

## Abstract

Attention deficit hyperactivity disorder (ADHD) is the most commonly diagnosed neurodevelopmental disorder in childhood. It is a heterogeneous disorder in terms of clinical presentation that is probably due to the frequent occurrence of comorbidity. Children with ADHD more frequently report sleep disorders (notably delayed sleep phase syndrome) and excessive daytime sleepiness (EDS) than typically developing children. The aim of this article is to propose a narrative review of the assessment of EDS in the context of ADHD with first a summary of the subjective and objective tools used to measure it. Secondly, perspectives in terms of electroencephalogram (EEG) markers and neurofeedback are proposed. Then, possibilities for new kinds of evaluation are discussed (virtual reality, ecological momentary assessment, etc.). Lastly, we discuss specific clinical situations with EDS in the context of ADHD as links with narcolepsy, the comorbidity with other psychiatric disorders, and the context of sluggish cognitive tempo.

## Introduction

Attention deficit hyperactivity disorder (ADHD) is the most commonly diagnosed neurodevelopmental disorder in childhood with a worldwide estimated prevalence of about 5% (1). It is characterized by inappropriate levels of inattention, impulsivity, and hyperactivity (2). Follow-up studies have documented the persistence of ADHD into adulthood in 50% to 65% of cases (3).

Sleep disorders have been extensively investigated in patients with ADHD, and their prevalence is reported to be in the range of 25–55% (4–6). Children with ADHD commonly exhibit additional mental health and neurodevelopmental comorbidities (7). Taurines R (8) introduces the notion of “developmental comorbidity” to highlight the importance of considering the age- and development-dependent occurrence of comorbidity in this disorder. Concerning the chronological order of occurrence, he posits that psychiatric conditions may be present before the appearance of first definite ADHD symptoms (“pre-comorbidity,” such as temperament factors, sleep disturbance, autism spectrum disorders, and atopic eczema). This work underlines the importance of sleep in the context of ADHD.

The most dominant sleep disturbance observed in patients with ADHD is delayed sleep phase syndrome (DSPS) associated with difficulties in falling asleep, difficulties in awakening, and/or excessive daytime sleepiness (EDS), characterized by difficulties in maintaining adequate alertness for daily activities with sleep occurring unintentionally or at inappropriate times almost daily.

Children with ADHD report EDS and more sleep problems than typically developing children (9). Other studies confirmed this subjective EDS (10–12) and notably described insufficient sleep on school days and during weekends and a higher level of EDS (13). Contrary to subjective data, there is significantly less evidence supporting group differences in objective measures of sleepiness (major findings in **Table 1**). Moreover, both meta-analyses of Cortese in 2006 and 2009 (9, 19) demonstrated that children with ADHD showed a tendency to be sleepier than controls during the daytime but without reaching pathological levels. Thus, while all these data point to the existence of objective EDS, the nature of this EDS remains to be determined. It might be a primary disorder or the consequence of other sleep disorders. Indeed, most studies with objective measures using the maintenance sleep latency test (MSLT) excluded patients with primary sleep disorders. An exception is the study by Prihodova (17), where 10 patients presented sleep-disordered breathing and 9 had periodic limb movements (PLMD) when asleep. Furthermore, the methodology used for the MSLT differed from one study to another (see **Table 1**). The mean age of the patients must also be taken into account, with younger patients in the work by Lecendreux (15) and older ones in the study by Golan (16). Indeed, a decrease in mean sleep latency with Tanner stages has been reported in healthy subjects. Moreover, even though patients were free of treatment and had stopped treatment at least 2 days before the test, there may have been an “after-effect” on sleepiness assessment. Since ADHD is a heterogeneous disorder, identification of its phenotypes would allow better understanding of its clinical diversity. Sleep and EDS are dimensions that may be involved in specific phenotypes.

**Table 1:** Major findings of MSLT in studies with ADHD children compared to control children.

Authors	Population	MSLT	Results
Palm et al. (14)	10 children with DAMP (deficit in attention, motor control, and perception) 18 control children 6–12 years	4X 30 min 10 am, 12 am, 2 pm, and 4 pm	Mean sleep latency DAMP vs. controls: 24.8 vs. 26.5 min (NS)
Lecendreux et al. (15)	26 ADHD children (*DSM IV*) 21 control children 5–10 years Medication free	4X 20 min 10 am, 12 am, 2 pm, and 4 pm	Mean sleep latency ADHD vs. controls: 16.7+/−5.4 min vs. 18.9+/− 3 (NS) Significant differences between groups for MSLT at 10 am, 12 am, and 2 pm
Golan et al. (16)	32 ADHD children (*DSM IV*), mean age: 12.4+/−4.6 years 29 control children, mean age: 12.0+/−3.6 years Medication free	5X 30 min 8 am, 10 am, 12 am, 2 am, and 4 pm	Mean sleep latency ADHD vs. controls: 21.9+/−2.6 min vs. 27.9+/− 1.85 min (p < .005) Significant differences between groups for MSLT at 8 am, 10 am, 2 am, and 4 pm
Prihodova et al. (17)	26 ADHD children (*DSM IV*) 26 control children 6–12 years Medication free	5X 20 min 10 am, 12 am, 2 am, 4 pm, and 6 pm	Mean sleep latency ADHD vs. controls: 16.1+/−3.4 min vs. 17.1+/− 2.4 min (NS) No significant difference between groups for MSLT
Wiebe et al. (18)	26 ADHD children (*DSM IV*) 56 control children 7–11 years	4X 20 min 10 am, 12 am, 2 pm, and 4 pm	Mean sleep latency ADHD vs. controls: 18.4+/−2.2 min vs. 17.5+/− 3.5 min (NS) No significant difference between groups for MSLT

MSLT, maintenance sleep latency test; DSM IV, Diagnostic and Statistical Manual of Mental Disorders version IV; ADHD, attention deficit hyperactivity disorder; NS, not significant.

The aim of this article is to propose a narrative review of the assessment of EDS in the context of ADHD with first a summary of the subjective and objective tools used to measure it. Secondly, perspectives in terms of electroencephalogram (EEG) markers and neurofeedback are proposed. Then, possibilities for new kinds of evaluation are discussed [virtual reality, ecological momentary assessment (EMA), etc.]. Lastly, we discuss specific clinical situations with EDS in the context of ADHD as links with narcolepsy, the comorbidity with other psychiatric disorders, and the context of sluggish cognitive tempo (SCT).

## Methods

We used the following search strategy. We considered papers examining the assessment of EDS in ADHD children. Only data published in English were included. We conducted a narrative review using the PubMed database up to June 2019 with the following keywords combination: “ADHD children” and (“sleepiness” or “excessive daytime sleepiness” or “hypoarousal”) and “assessment”. For perspectives, the search in PubMed used the following keywords: “ADHD children” and (“sleepiness” or “excessive daytime sleepiness” or “hypoarousal” or “EEG” or “arousal” or “vigilance”) or “neurofeedback”.

## Assessment of Sleepiness in Children

### Clinical Assessment

The clinical expression of EDS in children is very variable and differs from that exhibited by adults. Sleepiness can occur intermittently, often during passive activities such as reading and watching TV. At first, sleepy children may exhibit inattention, hyperactivity, or behavioral problems. This clinical presentation may mimic a patient with ADHD, which raises the issue of the differential diagnosis.

#### Clinical History

A clinical detailed history is crucial for the initial evaluation of a child with EDS (20, 21). First, the sleep history should include a review of the child’s wake/sleep schedule on weekdays, weekends, and during summer. It is important to report the child’s sleep habits and estimate his/her sleep quantity (total daily 24 h sleep time with naps), which may be compared and interpreted with the norms for age and overall level development (21). Secondly, the clinical presentation frequently provides enough data to suggest a sleep disorder. Symptoms should be screened for sleep-disordered breathing, circadian disorder, PLMD, narcolepsy symptoms, and medical causes of EDS (21). Finally, a detailed sleep diary recording at least 2 weeks of the child’s wake/sleep schedule confirms the information provided by the parents and the child. It is also important to probe the use of caffeine or other stimulants, and whether the child is taking a sedative. Sedentary habits such as watching TV, playing video games, and/or excessive snacking must be explored.

In the event of ADHD, it is important to explore the clinical history of the sleep symptoms and compare it with the prescription of stimulants. In fact, stimulants can induce insomnia but also can improve sleep disorders in the context of ADHD. Moreover, in a subgroup of children with ADHD taking methylphenidate, EDS increased a few hours after taking it. The study by Cockcroft K (22) showed that children with ADHD treated by methylphenidate demonstrated significantly higher levels of subjectively EDS 6 h after taking their stimulants both in the morning and in the afternoon.

#### Physical Examination

The physical examination is often normal in children with EDS. This examination can provide indications on sleep disorders such as sleep-disordered breathing in children, as in those with adenoid facies.

### Subjective Evaluation Tools

There is no specific questionnaire to assess sleepiness for children with ADHD. The questionnaires adapted for children can be used in patients with ADHD.

#### Self-Reported Sleepiness Questionnaires

Even if EDS is a frequent symptom in sleep disorders, validated tools in pediatric populations are lacking. Three clinical rating scales have been used to assess sleepiness specifically: the Stanford Sleepiness Scale (23), the Epworth Sleepiness Scale (ESS) (24), and the Pediatric Sleepiness Scale (11). The recent review by Benmedjahed (25) on the evaluation of sleepiness in children concluded that the ESS (24) or ESS versions modified for pediatric populations was the most frequently used measure of EDS. The ESS is the most commonly used measure of EDS. The eight questions assess sleep propensity in eight different daily-life activities. This questionnaire was validated in a population of adults with narcolepsy (26). Various modified versions of the EES are currently in use in pediatric populations with situations adapted to children. Melendres et al. (27) proposed a modified ESS (two items modified from the adult version to be more applicable to children), and Johns (28) proposed another modified version of this scale for children and adolescents called the ESS-CHAD. Scores range from 0 to 32. A score > 10 indicates the need for further evaluation.

The Pediatric Daytime Sleepiness Scale (PDSS) is a validated measure for assessing daytime sleepiness in children. It was developed to assess EDS in young school-age populations. It is an eight-item questionnaire evaluating subjective experiences of daytime sleepiness (items are scored from 0 to 4). Scores range from 0 to 32 (normal score <15, cutoff for sleepiness >20).

The Teacher’s Daytime Sleepiness Questionnaire (TSDQ) (29) is a teacher-reported measure. It is a six-item questionnaire rated on a three-point scale.

#### Self-Reported Sleep Questionnaires for Children

There are other questionnaires that assess sleep in children. In these questionnaires, some questions concern sleepiness. The most frequently used questionnaires are the following: the Sleep Disturbances Scale for children (30) (with the subscale excessive somnolence), the Sleep Disorder Inventory for children and adolescents (31), the Cleveland Adolescent Sleepiness Questionnaire (32), the Children’s Sleep Habits Questionnaire, and the Pediatric Sleep Questionnaire (PSQ) (12). The reviews by Moreira GA (33) and Benmedjahed (25) confirm the dearth of available validated measures for assessing EDS in pediatric populations.

### Objective Measures

#### Multiple Sleep Latency Test

The Multiple Sleep Latency Test (MSLT) is the recommended test to assess objective sleepiness both adults and children (32). This test assesses the time it takes to fall asleep (objective by the first epoch scored as sleep) during the daytime. The MSLT instructs the subject to fall asleep (protocol defined in AASM Practice Parameters for Clinical Use of MSLT, 2005) (34) while lying in bed in a dark and quiet room for five 20 min periods spaced at 2 h intervals. It is technically feasible and provides clear results in developmentally normal children aged 5 years and older (35–37), which showed that average MSLT sleep latency of 10 min or less is statistically significant for healthy children 5–16 years of age on bedtime and rise time schedules that allow at least 10 h of sleep per night.

As stated in these guidelines (36): “The MSLT, preceded by nocturnal PSG, is indicated in children suspected of having hypersomnia from causes other than narcolepsy to assess excessive sleepiness and to aid in differentiation from narcolepsy” (option level). Indeed, normative data indicate that children who were prepubertal or at early pubertal stages were less likely to fall asleep during the MSLT than older adolescents. These data suggest that the standard protocol may underestimate mild degrees of sleepiness (36). Thus, some researchers have modified the standard protocol by using 30 min nap opportunities instead of 20 min for prepubertal children, but more research is needed to confirm the impact of this increased time for nap opportunities (38, 39). In children with narcolepsy, the standard MSLT protocol appears adapted. The recent work from Pizza (40) exhibited that a mean sleep latency ≤8.2 min at the MSLT are valid and reliable markers for pediatric NT1 diagnosis. Nevertheless, no normative data for sleep latency have been found in the preschool population in whom daytime napping is customary.

#### Actigraphy

Actigraphy monitors rest/activity cycle by integrating the occurrence and degree of arm movement activity over time. It provides information about daytime activity, night/sleep activity (sleep patterns estimated by scoring algorithms) and activity circadian rhythm. This information can be useful in assessing sleep problems in adolescents with ADHD, especially to determine sufficient and insufficient sleep duration or stability or variability in sleep/wake patterns (41, 42). Martin-Martinez (43) propose a novel methodology for the automatic diagnosis of the combined type of ADHD based on nonlinear signal processing of actigraphy by combining a feature from the 24 h analysis, with features from the first part of sleep and the afternoon activity interval. Actigraphy cannot measure daytime sleepiness. The AASM recommendations suggest using actigraphy to assess pediatric patients with circadian rhythm sleep/wake disorder, to monitor total sleep time prior to testing with MSLT in pediatric patients with suspected central disorders of hypersomnolence and to estimate total sleep time in “adult” patients with suspected insufficient sleep syndrome. As it has been shown that children, especially boys, move during sleep more often than adults, actigraphy should be used with caution with the understanding that actigraphy has both strengths (multiday assessment, ability to detect sleep) and limitations (overestimation of wake during the sleep period) (44).

## Perspectives in Terms of Evaluation of Sleepiness With EEG Characteristics and Related Treatment

### EEG Characteristics

Sleepiness can be evaluated objectively by power density of waking EEG. The sleepier the subject, the more alpha (8–12 Hz) (45) and/or theta (4–8 Hz) (45, 46) EEG activity with eyes open will be evident, thus giving objective information about sleepiness. The evolution of theta/alpha power (6.25–9 Hz) of waking EEG across the 24 h day reflects the sleep homeostatic process (evolution of sleep pressure during 24 h) (47).

In children and adolescents with ADHD, the most dominant waking EEG features are increased power of slow waves and/or decreased power of fast waves recorded in resting state and over the fronto-central region in comparison with healthy children/adolescents [reviewed in (48–50)]. These EEG abnormalities could represent a cortical “slowing,” characterized by an increase in theta band activity (4–7 Hz) and by an increase in delta band activity (0–4 Hz) coupled with a decrease in beta band activity (14–30 Hz) [reviewed in (48–50)]. In adults with ADHD, the cortical “slowing” was maintained and was characterized by an increase in theta band activity in comparison with healthy adults (50).

This increase in theta band activity can reflect sleepiness (51) and/or cortical hypoarousal (52–54) both in children/adolescents and adults with ADHD, thereby confirming the hypoarousal model described above (55, 56). However, using the skin conductance level (SCL) as an unusual marker of drowsiness, Barry (57) suggested that alpha activity is the major band associated with arousal and not theta or beta activity in patients with ADHD. Clarke et al. (58) demonstrated that a subgroup with excess beta activity was not hyperaroused (assessed by SCL) and that the theta-to-beta ratio (TBR) was not associated with arousal.

Other studies have investigated the TBR [ratio of theta band (4–7 Hz) power divided by beta band (13–30 Hz) power [reviewed by (48, 49)]. In children/adolescents with ADHD, this ratio is abnormally higher than in healthy subjects. The meta-analysis of Snyder (59) demonstrated that TBR is remarkably robust with an effect size of 3.08 and a sensitivity and specificity greater than 90%. In view of these results, the Food and Drug Administration (FDA) approved the Neuropsychiatric EEG-Based Assessment Aid (NEBA) System (using a single electrode recording from central-midline location (Cz) and a ground electrode at the frontal-midline location (Fz) with eyes closed to obtain the TBR) as a diagnostic biomarker of ADHD in 2013. Recent studies did not confirm elevated TBR in adults with ADHD (60, 61) and suggest that it is age-dependent (50).

The review by Newson (50), which identified 65 studies in children and adults with ADHD, showed that the results differ greatly depending on the EEG power used (relative or absolute) and the recording conditions used (eyes open or closed). Consistency and validation scores in children were better in the eyes-closed condition with than with eyes open. Moreover, the studies analyzed absolute or relative power band, but the mean effect size was better with relative band than with absolute band (62, 63). Bussalb et al. (63) proposed a standardized method for calculating the TBR.

The heterogeneity in the EEG characteristics of these children could be related especially with the heterogeneity of the clinical profiles of ADHD. Already in 1996, Chabot and Serfontein (64) demonstrated three different EEG profiles in children with ADHD: one with a generalized increase in theta/alpha activity, another with an increase in alpha activity, and a third small group with an increase in beta activity.

Clarke et al. (65) identified three subgroups: the cortical hypoarousal group with elevated theta waves and decreased beta waves was coupled with symptoms of delinquent behaviors, the maturational lag group with elevated slow-wave (delta and theta) activity and decreased alpha waves, and the hyperarousal group with excessive beta activity was coupled with markers of ritualistic obsessive behaviors. These three subgroups were replicated, and an additional group was subsequently added (increased alpha and beta band power) (66).

Loo et al. (67), in a large sample of children, identified five subgroups of children with ADHD according to their resting EEG characteristics. Those with elevated slow-wave activity (delta and theta band) had higher levels of externalizing behaviors and cognitive deficits. Latent subgroups with elevated alpha and beta power had higher levels of internalizing behaviors, emotion dysregulation, and intact cognitive functioning. They demonstrated that there was no resting EEG abnormality that specifically characterizes children with ADHD or without ADHD and that there are age and gender differences in resting EEG characteristics (68). Boys with the combined subtype had higher theta power and lower alpha power than those with the inattentive type (68). Loo et al. (51) reported that the TBR was higher in the combined subtype than in the attentive subtype. Recently, Bussalb et al. (63) demonstrated two distinct subgroups in 363 children: one characterized by an elevated TBR and the other with a normal TBR.

The results analyzing resting state cortical activity in patients with ADHD are very heterogeneous, and the identification of distinct EEG subgroups is not consensual. Nevertheless, since EEG analysis is considered to be the gold standard to quantify sleepiness, evidence is accumulating of a subgroup of patients characterized by cortical hypoarousal.

In a different approach, the group of Hegerl (69, 70) using an automatic resting EEG classification of sleepiness [Vigilance Algorithm Leipzig software (VIGALL, seven EEG-vigilance stages)] demonstrated unstable arousal regulation in children and adults with ADHD. This arousal instability was characterized by a faster decline to the low EEG-vigilance stages and more fluctuations in their stages of vigilance, i.e., higher number of stage switches. A vigilant state instability [unstable vigilant state that fluctuates (71)] is observed in healthy sleep-deprived subjects: increasing sleep drive brings about escalating state instability in attention (the effort to recover and maintain attention increases), making neurobehavioral performance increasingly variable. This state instability could explain the higher sleepiness observed in patients with ADHD.

Despite years of research and the identification of clusters, quantitative EEG is not helpful in classifying ADHD patients and cannot be considered as a diagnostic biomarker for ADHD. However, the identification of EEG subgroups may help to optimize treatments (stimulant and non-stimulant treatment, neurofeedback, exercise, and dietary intervention) and/or measure the response to them. Indeed, stimulant treatment was more efficient in the hypoarousal subgroup [for review, see Kirkland (72)] or unstable alertness (70) and was not efficient in ADHD patients with a slowed individual alpha peak frequency (73).

### Treatment With Neurofeedback

Neurofeedback training (NFT) analyzes EEG activity and transforms it into instant feedback (visual and/or auditory signal) received by the user. By learning, the user self-regulates the amplitude of specific EEG activity. Neurofeedback is considered a non-pharmacological treatment option for ADHD [for review, see van Doren (74)].

The efficacy of NFB for children with ADHD is controversial. Meta-analyses published in the past decade have been contradictory (i.e., 75–78). For some authors like Thibault (75), the mechanisms involved raise questions. They suggest that neurofeedback could be an especially powerful form of placebo intervention, a kind of “superplacebo.” The most common NFT protocols in the treatment of ADHD are theta/beta training, and sensorimotor rhythm (SMR, increase activation at 12–15 Hz) and slow cortical potential (SCP) protocols. SCP neurofeedback has been compared to alpha-enhancement neurofeedback. Theta/beta training aims at reinforcing reductions in theta activity and increases in beta activity and could be more appropriate and efficient in the hypoarousal subgroup of patients with ADHD (76). SMR aims to decrease delta and beta activity. In light of the hyperarousal theory of insomnia, SMR reduces the symptoms of insomnia, especially the hyperarousal. SMR could be more appropriate and efficient in the hyperarousal subgroup of patients with ADHD. Bussalb et al. (63), who, in analyzing TBR, differentiated two distinct subgroups of ADHD patients, consider that neurofeedback should be divided into two subgroups: TBR downward and SMR upward. In all cases (diagnostic biomarker, prognostic biomarker, and/or biomarker of therapeutic response), more research is needed before EEG activity can be applied in clinical settings.

## Neuropsychological Markers: Links Between Attention and Sleepiness

### Attentional Process and Arousal Level

Arousal and attention are heterogeneous processes that interact with each other (77).

On one hand, attention corresponds to the appropriate allocation of processing resources to relevant stimuli. Attention is sub-divided into: (a) attentional orientation (the simple direction of attention to a particular stimulus); (b) selective (or focused) attention (giving attentional priority to one stimulus instead of another); (c) divided attention (dividing attention between two or more different stimuli); and (d) sustained attention (attending to one stimulus over an increasing period of time). On the other hand, arousal can be defined as the state of physiological reactivity, ranging on a continuum from sleep to alert wakefulness. Sleepiness increases the probability of the transition from wakefulness to sleep.

The arousal level controlled by homeostatic sleep pressure and the waking systems has repercussions on the attentional system, which modulates cognitive components such as executive functioning. The fundamental biological rhythm of sleep/wakefulness controlling the level of arousal is essential for maintaining optimal brain functions. Normal sleep ensures alert wakefulness, which is essential for appropriate decision-making and optimal adaptation to the environment, and is beneficial for global cognition (78).

The level of arousal can have an impact on cognitive functioning, in particular on attention and executive functions, *via* the modulation of the level of vigilance. Vigilance is a state of high efficiency of the central nervous system and is considered in a behavioral perspective as sufficient preparedness to detect and respond to critical events that occur rarely, and which are difficult to discriminate in extremely monotonous situations (e.g., detection tasks) (79). Vigilance could be the behavioral correlate of physiological alertness.

### Links With Executive Functions

This hypothesis echoes the model of attention proposed by Van Zomeren and Brouwer (80), which is widely used in neuropsychology and distinguishes an “intensity” component (vigilance/alert) and a “selectivity” component (selective attention and divided). In this model, the alert component refers to the ability to respond quickly and appropriately to the demands of the environment. It can be linked with the mobilization of energy so that the nervous system responds better. Tonic alert corresponds to a general state of arousal, which varies during the day. Phasic alert relates to the voluntary capacity to rapidly increase the general level of attention to anticipate an expected event. This vigilance/alert component modulates attention, which is a prerequisite to any other cognitive function. Thus, the influence of arousal level on cognitive functioning operates through the influence of the intensity component on component selectivity. This vigilance/alert component *via* modulation of attention also impacts executive functions. Executive functions govern all cognitive functions necessary to control and execute complex non-routine activities (81). Executive functions are a set of processes involving flexibility, decision-making, inhibition, planning, coordination, and control of thoughts and actions. Their main function is to facilitate the adaptation of the subject to the requirements and sudden fluctuations of the environment, especially when faced with new situations for which the action routines are no longer sufficient. Impairment of executive functioning results in disturbances in the complex activities of daily living.

### New Dimensional Tools to Assess the Impact of Sleepiness on Cognitive Functions

Impairments in sleep and alertness frequently occur in children with ADHD (19). Moreover, children and adolescents with ADHD often exhibit symptoms of inattention. Cognitive disorders affecting vigilance, attention, cognitive control, and executive functions are usually observed (82–85), leading to difficulties in performing complex daily life activities (86). Daily life is punctuated by a variety of complex activities related to different areas for children and adolescents (education, pedestrian safety), which all require adequate levels of alertness and attention.

Therefore, the clinical evaluation of sleepiness in ADHD could be achieved in the future by measuring the influence of arousal level on cognitive functioning (vigilance, attention, executive functions), including by measuring these functions when performing complex activities of daily living. The measurement of the level of sleepiness could be considered from a physiological to a psychological–behavioral sphere.

Neuropsychological assessment, notably involving vigilance, could be informative about the level of sleepiness. Calhoun, Fernandez-Mendoza, et al. (87) showed, in a large general population of young children, that parent-reported EDS was associated with impairment in cognitive and behavioral functioning. The Continuous Performance Test (CPT) is the most widely used task to assess vigilance, attention and impulsivity in children with ADHD (88). The KITAP (Attention Assessment Battery—Child Version) allows assessment of the attentional and executive performance of school-age children with validated standardized tests. The tests allow the evaluation of the “intensity” component of attention such as alertness and sustained attention, and the “selectivity” component such as selective attention and divided attention, as well as executive functions such as mental flexibility or inhibition.

An innovative approach could be to use the technology of virtual reality to determine the level of sleepiness as a function of cognitive alteration in ADHD children and adolescents immersed in the ecological tasks of everyday life. A study using a simulated driving task showed that not only the objective level of alertness but also inhibitory control deficits contribute independently to highway driving impairment in adults with ADHD (86). Virtual reality simulation is a technology that could allow the influence of arousal level on cognitive functioning (attention and executive functions) to be measured through vigilance and its impact on complex activities of daily life. In parallel, the monitoring of eyelid frequency and pupil diameter (eye-tracking system) or facial dynamic changes (89) could be promising (90).

Another innovative approach could be the use of EMA to evaluate daily and diurnal variations in sleepiness and repercussions on daily life such as in the emotional, cognitive, behavioral, social, and physical domains (91, 92) in ADHD.

## Excessive Daytime Sleepiness in Children With ADHD

The links between ADHD and sleep disorders remain unclear. While specific sleep disorders are a frequent comorbid condition associated with ADHD according to a categorical approach, they can also induce ADHD-like symptoms if considered dimensionally and are thought to be the consequence of EDS. Given the complexity of the interaction between ADHD and sleep, the team of Miano (93–95) identified five sleep phenotypes in ADHD: a sleep phenotype characterized by a hypo-arousal state “narcolepsy-like,” another associated with delayed sleep onset latency, a phenotype related with sleep-disordered breathing, another associated with restless legs syndrome and/or PLMD, and a phenotype related to epilepsy/or EEG interictal discharges.

### Links With Narcolepsy

Narcolepsy, which is characterized by EDS and abnormal REM sleep, is a chronic sleep disorder caused by a deficiency of hypocretin/orexin-producing neurons within the lateral hypothalamus (type 1 narcolepsy with cataplexy). In type 2 narcolepsy, where cataplexy is absent and orexin levels normal, the physiopathology is unknown. The “narcolepsy-like” phenotype (ADHD and hypersomnia) described by Miano could be part of a subtype of type 2 narcolepsy (96). A body of evidence reinforces the links between ADHD and narcolepsy, and ADHD symptoms are more frequent in children (97) or adults (98) with narcolepsy than in control subjects. A systematic review demonstrated that the prevalence of ADHD symptoms in narcolepsy was >30% (99).

In addition, adult narcoleptics present more childhood ADHD than controls (100, 101). Using MSLT or EEG features, some authors have hypothesized the existence of a dysfunction in arousal in the pathophysiology in ADHD (15, 65, 100). A physiopathological model for ADHD involves a deficit in arousal (hypo-arousal model). Children with ADHD have greater difficulty in falling asleep, awakening, and/or maintaining adequate daytime alertness than control children, and use hyperactivity as a strategy to stay awake and alert in order to counteract the tendency to fall asleep (55, 56).

Moreover, high levels of EDS and shorter REM latency are classically both potential signs of narcolepsy (102). In the study by Diaz-Roman (10), polysomnographic recordings showed that children with ADHD presented significantly greater general sleep problems than control subjects and shorter REM latency in the ADHD group. Nevertheless, other studies failed to find differences in REM latency between children with and without ADHD in polysomnography (PSG) (9, 103). A recent study found that serum orexin A levels were significantly lower in drug-naive children with ADHD, especially in the attention deficit dominant subgroup (104). As suggested by Cortese (105), orexin neurons located in the perifornical and dorsomedial hypothalamic areas (implicated in arousal) could be hypoactivated, while those located in the lateral hypothalamus (involved in reward processing, stimulating feeding, and other reward-seeking behaviors) could be overactivated in patients with ADHD.

### Specific Clinical Situations

“Sluggish cognitive tempo” (SCT) is a behavioral construct characterized by a set of symptoms as sluggish, under-motivated, lethargic, slowed, and/or forgetful behavior (106, 107). This condition is strongly associated with ADHD (notably with the inattentive subtype), although the meta-analysis by Becker et al. (106) identified SCT symptoms that were distinguishable from ADHD inattentive symptoms. Some authors hypothesized that SCT symptoms are specifically related with sleep problems and EDS (106, 108, 109). SCT symptoms were found to overlap with EDS in patients with ADHD but were distinct from sleep problems. Objective assessments of EDS may help to better phenotype these subjects and to propose other therapeutic strategies, to the extent that this condition predicts non-response or poorer response to methylphenidate in children with ADHD (110). Pharmacological treatment such as wakefulness drugs might be more indicated in this subgroup of patients.

### Comorbidity

Moreover, SCT is strongly associated with internalizing symptoms, especially depressive symptoms. It is important to evaluate sleep in the context of comorbidity (internalizing and externalizing disorders), as mentioned by Spruyt (111) and by Diaz-Roman (10). Comorbid conditions may also play a role in EDS in children. Diaz-Roman (10) found significant correlations between psychopathology and sleep measures with a significant correlation between scores on the Child Behavior Checklist (a questionnaire completed by the parents which evaluates internalizing and externalizing problems) and EDS and general sleep problems. This finding was in line with previous data supporting the link between sleep problems and children with ADHD with comorbid anxiety and depression (112), and with a study by Mulraney M et al. (113) who reported links between emotional problems and sleep disturbances in ADHD. When assessing sleep in children with ADHD, it is also necessary to evaluate other psychiatric comorbidities, notably internalizing problems such as depression and anxiety that seem to play a role in the expression of EDS in these children.

In conclusion, ADHD is a heterogeneous disorder in terms of its clinical presentation and underlying physiopathological mechanisms. EDS, a usual complaint in children with ADHD, seems to be a pertinent clinical dimension. Thus, the identification of a specific “sleepy ADHD” phenotype appears to be both a clinical and objective maker. The identification of EEG subgroups would help in defining various phenotypes and optimizing treatments. More studies are needed in children with ADHD to assess EDS. The interactions between EDS and the attentional process and executive functions should also be probed.

## Author Contributions

SB wrote sections *Introduction* and *Assessment of Sleepiness in Children* of the manuscript. JT wrote section *Perspectives in Terms of Evaluation of Sleepiness With EEG Characteristics and Related Treatment* of the manuscript. PS wrote section *Neuropsychological Markers: Links Between Attention and Sleepiness* of the manuscript. PP wrote the *Introduction* and did a rereading of the manuscript. All authors contributed to manuscript revision, read and approved the submitted version.

## Conflict of Interest

The authors declare that the research was conducted in the absence of any commercial or financial relationships that could be construed as a potential conflict of interest.

## References

[1] Polanczyk GV Willcutt EG Salum GA Kieling C Rohde LA ADHD prevalence estimates across three decades: an updated systematic review and meta-regression analysis. Int J Epidemiol (2014) 43(2):434–42. 10.1093/ije/dyt261 PMC4817588 24464188

[2] APA Diagnostic and Statistical Manual of Mental Disorders; DSM-5. 5th ed. Washington, DC: American Psychiatric Association (2013).

[3] Lara C Fayyad J de Graaf R Kessler RC Aguilar-Gaxiola S Angermeyer M Childhood predictors of adult attention-deficit/hyperactivity disorder: results from the World Health Organization World Mental Health Survey Initiative. Biol Psychiatry (2009) 65(1):46–54. 10.1016/j.biopsych.2008.10.005 19006789 PMC2629074

[4] Hvolby A Associations of sleep disturbance with ADHD: implications for treatment. Atten Defic Hyperact Disord (2015) 7(1):1–18. 10.1007/s12402-014-0151-0 PMC4340974 25127644

[5] Yoon SY Jain U Shapiro C Sleep in attention-deficit/hyperactivity disorder in children and adults: past, present, and future. Sleep Med Rev (2012) 16(4):371–88. 10.1016/j.smrv.2011.07.001 22033171

[6] Cortese S Brown TE Corkum P Gruber R O'Brien LM Stein M Assessment and management of sleep problems in youths with attention-deficit/hyperactivity disorder. J Am Acad Child Adolesc Psychiatry (2013) 52(8):784–96. 10.1016/j.jaac.2013.06.001 23880489

[7] Larson K Russ SA Kahn RS Halfon N Patterns of comorbidity, functioning, and service use for US children with ADHD, 2007. Pediatrics (2011) 127(3): 462–70. 10.1542/peds.2010-0165 PMC3065146 21300675

[8] Taurines R Schmitt J Renner T Conner AC Warnke A Romanos M Developmental comorbidity in attention-deficit/hyperactivity disorder. Atten Defic Hyperact Disord (2010) 2(4):267–89. 10.1007/s12402-010-0040-0 21432612

[9] Cortese S Faraone SV Konofal E Lecendreux M Sleep in children with attention-deficit/hyperactivity disorder: meta-analysis of subjective and objective studies. J Am Acad Child Adolesc Psychiatry (2009) 48(9):894–908. 10.1097/CHI.0b013e3181ae09c9 19625983

[10] Diaz-Roman A Buela-Casal G Shorter REM latency in children with attention-deficit/hyperactivity disorder. Psychiatry Res (2019) 278:188–93. 10.1016/j.psychres.2019.06.012 31207456

[11] Drake C Nickel C Burduvali E Roth T Jefferson C Pietro B The pediatric daytime sleepiness scale (PDSS): sleep habits and school outcomes in middle-school children. Sleep (2003) 26(4):45–58. 12841372

[12] Chervin RD Hedger K Dillon JE Pituch KJ Pediatric sleep questionnaire (PSQ): validity and reliability of scales for sleep-disordered breathing, snoring, sleepiness, and behavioral problems. Sleep Med (2000) 1(1):21 –32. 10.1016/S1389-9457(99)00009-X 10733617

[13] Becker SP Epstein JN Tamm L Tilford AA Tischner CM Isaacson PA shortened sleep duration causes sleepiness, inattention, and oppositionality in adolescents with attention-deficit/hyperactivity disorder: findings from a crossover sleep restriction/extension study. J Am Acad Child Adolesc Psychiatry (2019) 58(4):433–42. 10.1016/j.jaac.2018.09.439 PMC6441371 30768404

[14] Palm L Persson E Bjerre I Elmqvist D Blennow G Sleep and wakefulness in preadolescent children with deficits in attention, motor control and perception. Acta Paediatr (1992) 81(8):618–24. 10.1111/j.1651-2227.1992.tb12313.x 1392387

[15] Cortese S Konofal E Yateman N Mouren MC Lecendreux M Sleep and alertness in children with attention-deficit/hyperactivity disorder: a systematic review of the literature. Sleep (2006) 29(4):504–11. 16676784

[16] Prihodova I Paclt I Kemlink D Skibova J Ptacek R Nevsimalova S Sleep disorders and daytime sleepiness in children with attention-deficit/hyperactivity disorder: a two-night polysomnographic study with a multiple sleep latency test. Sleep Med (2010) 11(9):922–8. 10.1016/j.sleep.2010.03.017 20817551

[17] Lecendreux M Konofal E Bouvard M Falissard B Mouren-Simeoni MC Sleep and alertness in children with ADHD. J Child Psychol Psychiatry (2000) 41(6):803–12. 10.1111/1469-7610.00667 11039692

[18] Wiebe S Carrier J Frenette S Gruber R Sleep and sleepiness in children with attention deficit/hyperactivity disorder and controls. J Sleep Res (2013) 22(1):41–9. 10.1111/j.1365-2869.2012.01033.x 22762354

[19] Golan N Shahar E Ravid S Pillar G Sleep disorders and daytime sleepiness in children with attention-deficit/hyperactive disorder. Sleep (2004) 27(2):261–6. 10.1093/sleep/27.2.261 15124720

[20] Palm L Persson E Elmqvist D Blennow G Sleep and wakefulness in normal preadolescent children. Sleep (1989) 12(4):299 –308. 10.1093/sleep/12.4.299 2762685

[21] Kothare SV Pomeroy SL Pediatric sleep medicine in 2008: state-of-the-art. Curr Opin Pediatr (2008) 20(6):639–40. 10.1097/MOP.0b013e328317ee99 19023916

[22] Hoban TF Chervin RD Assessment of sleepiness in children. Semin Pediatr Neurol (2001) 8(4):216–28. 10.1053/spen.2001.29043 11768784

[23] Cockcroft K Ashwal J Bentley A Sleep and daytime sleepiness in methylphenidate medicated and un-medicated children with attention-deficit/hyperactivity disorder (ADHD). Afr J Psychiatry (Johannesbg) (2009) 12(4):27–59. 10.4314/ajpsy.v12i4.49043 20033109

[24] Hoddes E Zarcone V Smythe H Phillips R Dement WC Quantification of sleepiness: a new approach. Psychophysiology (1973) 10(4):431–6. 10.1111/j.1469-8986.1973.tb00801.x 4719486

[25] Johns MW A new method for measuring daytime sleepiness: the Epworth sleepiness scale. Sleep (1991) 14(6):540–5. 10.1093/sleep/14.6.540 1798888

[26] Benmedjahed K Wang YG Lambert J Evans C Hwang S Black J Assessing sleepiness and cataplexy in children and adolescents with narcolepsy: a review of current patient-reported measures. Sleep Med (2017) 32:14–39. 10.1016/j.sleep.2016.12.020 28366326

[27] Melendres MC Lutz JM Rubin ED Marcus CL Daytime sleepiness and hyperactivity in children with suspected sleep-disordered breathing. Pediatrics (2004) 114(3):768–75. 10.1542/peds.2004-0730 15342852

[28] Johns MW Sensitivity and specificity of the multiple sleep latency test (MSLT), the maintenance of wakefulness test and the epworth sleepiness scale: failure of the MSLT as a gold standard. J Sleep Res (2000) 9(1):5–11. 10.1046/j.1365-2869.2000.00177.x 10733683

[29] Johns M The assessment of sleepiness in children and adolescents. Sleep Biol Rhythm (2015) 13(Suppl. 1):97.

[30] Owens JA Maxim R Nobile C McGuinn M Msall M Parental and self-report of sleep in children with attention-deficit/hyperactivity disorder. Arch Pediatr Adolesc Med (2000) 154(6):549–55. 10.1001/archpedi.154.6.549 10850500

[31] Bruni O Ottaviano S Guidetti V Romoli M Innocenzi M Cortesi F The Sleep Disturbance Scale for Children (SDSC). Construction and validation of an instrument to evaluate sleep disturbances in childhood and adolescence. J Sleep Res (1996) 5(4):251–61. 10.1111/j.1365-2869.1996.00251.x 9065877

[32] Luginbuehl M Kohler WC Screening and evaluation of sleep disorders in children and adolescents. Child Adolesc Psychiatr Clin N Am (2009) 18(4):825–38. 10.1016/j.chc.2009.04.012 19836690

[33] Spilsbury JC The Cleveland adolescent sleepiness questionnaire: a new measure to assess excessive daytime sleepiness in adolescents. J Clin Sleep Med (2007) 3(6):603–12. 10.1037/t65526-000 PMC2045721 17993042

[34] Moreira GA Pradella-Hallinan M Sleepiness in children: an update. Sleep Med Clin (2017) 12(3):407–13. 10.1016/j.jsmc.2017.03.013 28778238

[35] Littner MR Kushida C Wise M Davila DG Morgenthaler T Lee-Chiong T Practice parameters for clinical use of the multiple sleep latency test and the maintenance of wakefulness test. Sleep (2005) 28(1):113–21. 10.1093/sleep/28.1.113 15700727

[36] Aurora RN Lamm CI Zak RS Kristo DA Bista SR Rowley JA Practice parameters for the non-respiratory indications for polysomnography and multiple sleep latency testing for children. Sleep (2012) 35(11):1467–73. 10.5665/sleep.2190 PMC3466793 23115395

[37] Aurora RN Kristo DA Bista SR Rowley JA Zak RS Casey KR The treatment of restless legs syndrome and periodic limb movement disorder in adultsan update for 2012: practice parameters with an evidence-based systematic review and meta-analyses: an American Academy of Sleep Medicine Clinical Practice Guideline. Sleep (2012) 35(8):1039–62. 10.5665/sleep.1986 PMC3397811 22851801

[38] Fallone G Seifer R Acebo C Carskadon MA How well do school-aged children comply with imposed sleep schedules at home? Sleep (2002) 25(7):739–45. 10.1093/sleep/25.7.739 12405609

[39] Gozal D Wang M Pope DW Jr. Objective sleepiness measures in pediatric obstructive sleep apnea. Pediatrics (2001) 108(3):693–7. 10.1542/peds.108.3.693 11533338

[40] Pizza F Barateau L Jaussent I Vandi S Antelmi E Mignot E Validation of multiple sleep latency test for the diagnosis of pediatric narcolepsy type 1. Neurology (2019) 93(11):e1034–44. 10.1212/WNL.0000000000008094 31405906

[41] Becker SP Langberg JM Eadeh HM Isaacson PA Bourchtein E Sleep and daytime sleepiness in adolescents with and without ADHD: differences across ratings, daily diary, and actigraphy. J Child Psychol Psychiatry (2019) 60(9):1021–31. 10.1111/jcpp.13061 PMC6692210 31032953

[42] Langberg JM Breaux RP Cusick CN Green CD Smith ZR Molitor SJ Intraindividual variability of sleep/wake patterns in adolescents with and without attention-deficit/hyperactivity disorder. J Child Psychol Psychiatry (2019) 60(11):1219–29. 10.1111/jcpp.13082 PMC6800768 31231801

[43] Martin-Martinez D Casaseca-de-la-Higuera P Alberola-Lopez S Andres-de-Llano J Lopez-Villalobos JA Ardura-Fernandez J Nonlinear analysis of actigraphic signals for the assessment of the attention-deficit/hyperactivity disorder (ADHD). Med Eng Phys (2012) 34(9):1317–29. 10.1016/j.medengphy.2011.12.023 22297088

[44] Meltzer LJ Wong P Biggs SN Traylor J Kim JY Bhattacharjee R Validation of actigraphy in middle childhood. Sleep (2016) 39(6):1219–24. 10.5665/sleep.5836 PMC4863209 27091520

[45] Akerstedt T Gillberg M Subjective and objective sleepiness in the active individual. Int J Neurosci (1990) 52(1–2):29 –37. 10.3109/00207459008994241 2265922

[46] Strijkstra AM Beersma DG Drayer B Halbesma N Daan S Subjective sleepiness correlates negatively with global alpha (8-12 Hz) and positively with central frontal theta (4-8 Hz) frequencies in the human resting awake electroencephalogram. Neurosci Lett (2003) 340(1):17–20. 10.1016/S0304-3940(03)00033-8 12648748

[47] Cajochen C Brunner DP Krauchi K Graw P Wirz-Justice A Power density in theta/alpha frequencies of the waking EEG progressively increases during sustained wakefulness. Sleep (1995) 18(10):890–4. 10.1093/sleep/18.10.890 8746397

[48] Newson JJ Thiagarajan TC EEG Frequency Bands in psychiatric disorders: a review of resting state studies. Front Hum Neurosci (2018) 12:521. 10.3389/fnhum.2018.00521 30687041 PMC6333694

[49] Loo SK Makeig S Clinical utility of EEG in attention-deficit/hyperactivity disorder: a research update. Neurotherapeutics (2012) 9(3):569–87. 10.1007/s13311-012-0131-z PMC3441927 22814935

[50] Satterfield JH Cantwell DP Saul RE Yusin A Intelligence, academic achievement, and EEG abnormalities in hyperactive children. Am J Psychiatry (1974) 131(4):391–5. 4814906

[51] Mann CA Lubar JF Zimmerman AW Miller CA Muenchen RA Quantitative analysis of EEG in boys with attention-deficit-hyperactivity disorder: controlled study with clinical implications. Pediatr Neurol (1992) 8(1):30–6. 10.1016/0887-8994(92)90049-5 1558573

[52] Lubar JF Discourse on the development of EEG diagnostics and biofeedback for attention-deficit/hyperactivity disorders. Biofeedback Self Regul (1991) 16(3):201–25. 10.1007/BF01000016 1932259

[53] Brown TE McMullen WJ Jr. Attention deficit disorders and sleep/arousal disturbance. Ann N Y Acad Sci (2001) 931:271–86. 10.1111/j.1749-6632.2001.tb05784.x 11462746

[54] Weinberg WA Harper CR Brumback RA Neuroanatomic substrate of developmental specific learning disabilities and select behavioral syndromes. J Child Neurol (1995) 10 Suppl 1:S78–80. 10.1177/08830738950100S115 7538526

[55] Barry RJ Clarke AR Johnstone SJ McCarthy R Selikowitz M Electroencephalogram theta/beta ratio and arousal in attention-deficit/hyperactivity disorder: evidence of independent processes. Biol Psychiatry (2009) 66(4):398–401. 10.1016/j.biopsych.2009.04.027 19500774

[56] Clarke AR Barry RJ Dupuy FE McCarthy R Selikowitz M Johnstone SJ Excess beta activity in the EEG of children with attention-deficit/hyperactivity disorder: a disorder of arousal? Int J Psychophysiol (2013) 89(3):31–49. 10.1016/j.ijpsycho.2013.04.009 23619205

[57] Snyder SM Hall JR A meta-analysis of quantitative EEG power associated with attention-deficit hyperactivity disorder. J Clin Neurophysiol (2006) 23(5):440–55. 10.1097/01.wnp.0000221363.12503.78 17016156

[58] Markovska-Simoska S Pop-Jordanova N Quantitative EEG in children and adults with attention deficit hyperactivity disorder: comparison of absolute and relative power spectra and Theta/Beta ratio. Clin EEG Neurosci (2017) 48(1):20–32. 10.1177/1550059416643824 27170672

[59] Saad JF Kohn MR Clarke S Lagopoulos J Hermens DF Is the Theta/Beta EEG marker for ADHD inherently Flawed? J Atten Disord (2018) 22(9):815–26. 10.1177/1087054715578270 25823742

[60] Boutros N Fraenkel L Feingold A A four-step approach for developing diagnostic tests in psychiatry: EEG in ADHD as a test case. J Neuropsychiatry Clin Neurosci (2005) 17(4):455–64. 10.1176/jnp.17.4.455 16387983

[61] Bussalb A Collin S Barthelemy Q Ojeda D Bioulac S Blasco-Fontecilla H Is there a cluster of high theta-beta ratio patients in attention deficit hyperactivity disorder? Clin Neurophysiol (2019) 130(8):1387–96. 10.1016/j.clinph.2019.02.021 31176621

[62] Chabot RJ Serfontein G Quantitative electroencephalographic profiles of children with attention deficit disorder. Biol Psychiatry (1996) 40(10):951–63. 10.1016/0006-3223(95)00576-5 8915554

[63] Clarke AR Barry RJ McCarthy R Selikowitz M Excess beta activity in children with attention-deficit/hyperactivity disorder: an atypical electrophysiological group. Psychiatry Res (2001) 103(2–3):205–18. 10.1016/S0165-1781(01)00277-3 11549408

[64] Clarke AR Barry RJ Dupuy FE Heckel LD McCarthy R Selikowitz M Behavioural differences between EEG-defined subgroups of children with Attention-Deficit/Hyperactivity Disorder. Clin Neurophysiol (2011) 122(7):1333–41. 10.1016/j.clinph.2010.12.038 21247797

[65] Loo SK McGough JJ McCracken JT Smalley SL Parsing heterogeneity in attention-deficit hyperactivity disorder using EEG-based subgroups. J Child Psychol Psychiatry (2018) 59(3):223–31. 10.1111/jcpp.12814 PMC5812789 28921526

[66] Dupuy FE Barry RJ Clarke AR McCarthy R Selikowitz M Sex differences between the combined and inattentive types of attention-deficit/hyperactivity disorder: an EEG perspective. Int J Psychophysiol (2013) 89(3):320–7. 10.1016/j.ijpsycho.2013.04.004 23603052

[67] Strauss M Ulke C Paucke M Huang J Mauche N Sander C Brain arousal regulation in adults with attention-deficit/hyperactivity disorder (ADHD). Psychiatry Res (2018) 261:102–8. 10.1016/j.psychres.2017.12.043 29291475

[68] Sander C Arns M Olbrich S Hegerl U EEG-vigilance and response to stimulants in paediatric patients with attention deficit/hyperactivity disorder. Clin Neurophysiol (2010) 121(9):1511–8. 10.1016/j.clinph.2010.03.021 20382071

[69] Doran SM Van Dongen HP Dinges DF Sustained attention performance during sleep deprivation: evidence of state instability. Arch Ital Biol (2001) 139(3):253–67. 11330205

[70] Kirkland AE Holton KF Measuring treatment response in pharmacological and lifestyle interventions using electroencephalography in ADHD: a review. Clin EEG Neurosci (2019) 50(4):256–66. 10.1177/1550059418817966 30626211

[71] Arns M Gunkelman J Breteler M Spronk D EEG phenotypes predict treatment outcome to stimulants in children with ADHD. J Integr Neurosci (2008) 7(3):421–38. 10.1142/S0219635208001897 18988300

[72] Van Doren J Arns M Heinrich H Vollebregt MA Strehl U K.L S Sustained effects of neurofeedback in ADHD: a systematic review and meta-analysis. Eur Child Adolesc Psychiatry (2019) 28(3):293–305. 10.1007/s00787-018-1121-4 29445867 PMC6404655

[73] Thibault RT Lifshitz M Raz A Neurofeedback or neuroplacebo? Brain (2017) 140(4):862–4. 10.1093/brain/awx033 28375458

[74] Arns M Heinrich H Strehl U Evaluation of neurofeedback in ADHD: the long and winding road. Biol Psychol (2014) 95:108–15. 10.1016/j.biopsycho.2013.11.013 24321363

[75] Coull JT Neural correlates of attention and arousal: insights from electrophysiology, functional neuroimaging and psychopharmacology. Prog Neurobiol (1998) 55(4):343–61. 10.1016/S0301-0082(98)00011-2 9654384

[76] Sagaspe P Taillard J Akerstedt T Bayon V Espié S Chaumet G Extended driving impairs nocturnal driving performances. PloS One (2008) 3(10):e34–93. 10.1371/journal.pone.0003493 PMC2566807 18941525

[77] Mackworth JF Vigilance, arousal, and habituation. Psychol Rev (1968) 75(4):308–22. 10.1037/h0025896 4875885

[78] Van Zomeren AH Brouwer WH New York: Oxford University Press (1994).

[79] Rabbitt P Methodology of frontal and executive function. Hove, England: Psychology Press/Erlbaum (UK) Taylor & Francis (1997).

[80] Barkley RA Grodzinsky G DuPaul GJ Frontal lobe functions in attention deficit disorder with and without hyperactivity: a review and research report. J Abnormal Child Psychol (1992) 20(2):163–88. 10.1007/BF0091654 1593025

[81] Schachar R Mota VL Logan GD Tannock R Klim P Confirmation of an inhibitory control deficit in attention-deficit/hyperactivity disorder. J Abnormal Child Psychol (2000) 28(3):227–35. 10.1023/a:1005140103162 10885681

[82] Nigg JT Neuropsychologic theory and findings in attention-deficit/hyperactivity disorder: the state of the field and salient challenges for the coming decade. Biol Psychiatry (2005) 57(11):1424–35. 10.1016/j.biopsych.2004.11.011 15950017

[83] Lecendreux M Cortese S Sleep problems associated with ADHD: a review of current therapeutic options and recommendations for the future. Expert Rev Neurother (2007) 7(12):1799–806. 10.1586/14737175.7.12.1799 18052772

[84] Bioulac S Sagaspe P Micoulaud-Franchi JA Altena E Taillard J Schröder C Objective level of alertness and inhibitory control predict highway driving impairment in adults with ADHD. J Atten Disord (2016). 10.1177/1087054716633751 27009924

[85] Calhoun SL Mendoza JF Vgontzas AN Mayes SD Tsaoussoglou M Rodriguez-Munoz A Learning, attention/hyperactivity, and conduct problems as sequelae of excessive daytime sleepiness in a general population study of young children. Sleep (2012) 35(5):627–32. 10.5665/sleep.1818 PMC3321421 22547888

[86] Huang-Pollock CL Karalunas SL Tam H Moore AN Evaluating vigilance deficits in ADHD: a meta-analysis of CPT performance. J Abnorm Psychol (2012) 121(2):360–71. 10.1037/a0027205 PMC3664643 22428793

[87] Poursadeghiyan M Mazloumi A Nasl Saraji G Niknezhad A Akbarzadeh A Ebrahimi MH Determination the levels of subjective and observer rating of drowsiness and their associations with facial dynamic changes. Iran J Public Health (2017) 46(1):93–102. 28451534 PMC5401941

[88] Rhle KH Franke KJ Nilius G [Microsleep, sleepiness and driving performance in patients with sleep apnoea syndrome]. Pneumologie (2008) 62(10):595 –601. 10.1055/s-2008-1038203 18711696

[89] Kaplan KA Talavera DC Harvey AG Rise and shine: a treatment experiment testing a morning routine to decrease subjective sleep inertia in insomnia and bipolar disorder. Behav Res Ther (2018) 111:106–12. 10.1016/j.brat.2018.10.009 30399503

[90] Dong L Gumport NB Martinez AJ Harvey AG Is improving sleep and circadian problems in adolescence a pathway to improved health? A Mediation Anal J Consult Clin Psychol (2019) 87(9):757–71. 10.1037/ccp0000423 PMC6690778 31246052

[91] Miano S Amato N Foderaro G Pezzoli V Ramelli GP Toffolet L Sleep phenotypes in attention deficit hyperactivity disorder. Sleep Med (2019) 60:123–31. 10.1016/j.sleep.2018.08.026 30377038

[92] Miano S Parisi P Villa MP The sleep phenotypes of attention deficit hyperactivity disorder: the role of arousal during sleep and implications for treatment. Med Hypotheses (2012) 79(2):147–53. 10.1016/j.mehy.2012.04.020 22608760

[93] Miano S Peraita-Adrados R Pediatric insomnia: clinical, diagnosis, and treatment. Rev Neurol (2014) 58(1):35 –42. 10.33588/rn.5801.2013398 24343539

[94] Ito W Komada Y Okajima I Inoue Y Excessive daytime sleepiness in adults with possible attention deficit/hyperactivity disorder (ADHD): a web-based cross-sectional study. Sleep Med (2017) 32:4 –9. 10.1016/j.sleep.2016.04.008 28366340

[95] Lecendreux M Lavault S Lopez R Inocente CO Konofal E Cortese S Attention-Deficit/Hyperactivity Disorder (ADHD) symptoms in pediatric narcolepsy: a cross-sectional study. Sleep (2015) 38(8):1285–95. 10.5665/sleep.4910 PMC4507734 26118560

[96] Filardi M Pizza F Tonetti L Antelmi E Natale V Plazzi G Attention impairments and ADHD symptoms in adult narcoleptic patients with and without hypocretin deficiency. PloS One (2017) 12(8):e0182085. 10.1371/journal.pone.0182085 28763482 PMC5538711

[97] Kim MJ Park I Lim MH Paik KC Cho S Kwon HJ Prevalence of attention-deficit/hyperactivity disorder and its comorbidity among korean children in a community population. J Korean Med Sci (2017) 32(3):401–6. 10.3346/jkms.2017.32.3.401 PMC5290097 28145641

[98] Modestino EJ Winchester J A retrospective survey of childhood ADHD symptomatology among adult narcoleptics. J Atten Disord (2013) 17(7):574–82. 10.1177/1087054713480033 23548870

[99] Lopez R Micoulaud-Franchi JA Camodeca L Gachet M Jaussent I Dauvilliers Y Association of inattention, hyperactivity, and hypersomnolence in two clinic-based adult cohorts. J Atten Disord (2018) 1087054718775826. 10.1177/1087054718775826 29771183

[100] Sateia MJ American Academy of Sleep Medicine, International Classification of Sleep Disorders 3rd ed. Darien, IL: American Academy of Sleep Medicine (2014).

[101] Diaz-Roman A Hita-Yanez E Buela-Casal G Sleep characteristics in children with attention deficit hyperactivity disorder: systematic review and meta-analyses. J Clin Sleep Med (2016) 12(5):747–56. 10.5664/jcsm.5810 PMC4865562 26951416

[102] Baykal S Albayrak Y Durankus F Guzel S Abbak O Potas N Decreased serum orexin a levels in drug-naive children with attention deficit and hyperactivity disorder. Neurol Sci (2019) 40(3):593 –602. 10.1007/s10072-018-3692-8 30617449

[103] Cortese S Konofal E Lecendreux M Alertness and feeding behaviors in ADHD: does the hypocretin/orexin system play a role? Med Hypotheses (2008) 71(5):770–5. 10.1016/j.mehy.2008.06.017 18678446

[104] Becker SP Leopold DR Burns GL Jarrett MA Langberg JM Marshall SA The Internal, external, and diagnostic validity of sluggish cognitive tempo: a meta-analysis and critical review. J Am Acad Child Adolesc Psychiatry (2016) 55(3):163–78. 10.1016/j.jaac.2015.12.006 PMC4764798 26903250

[105] Koriakin TA Mahone EM Jacobson LA Sleep difficulties are associated with parent report of sluggish cognitive tempo. J Dev Behav Pediatr (2015) 36(9):717–23. 10.1097/DBP.0000000000000224 PMC5080837 26468939

[106] Becker SP Marshall SA McBurnett K Sluggish cognitive tempo in abnormal child psychology: an historical overview and introduction to the special section. J Abnorm Child Psychol (2014) 42(1):1–6. 10.1007/s10802-013-9825-x 24272365

[107] Langberg JM Becker SP Dvorsky MR Luebbe AM Are sluggish cognitive tempo and daytime sleepiness distinct constructs? Psychol Assess (2014) 26(2):586–97. 10.1037/a0036276 24611789

[108] Froehlich TE Becker SP Nick TG Brinkman WB Stein MA Peugh J Sluggish cognitive tempo as a possible predictor of methylphenidate response in children with ADHD: a randomized controlled trial. J Clin Psychiatry (2018) 79(2). 10.4088/JCP.17m11553 PMC6558969 29489078

[109] Spruyt K Gozal D Sleep disturbances in children with attention-deficit/hyperactivity disorder. Expert Rev Neurother (2011) 11(4):565–77. 10.1586/ern.11.7 PMC3129712 21469929

[110] Mayes SD Calhoun SL Bixler EO Vgontzas AN Mahr F Hillwig-Garcia J ADHD subtypes and comorbid anxiety, depression, and oppositional-defiant disorder: differences in sleep problems. J Pediatr Psychol (2009) 34(3):328–37. 10.1093/jpepsy/jsn083 PMC2722128 18676503

[111] Mulraney M Giallo R Lycett K Mensah F Sciberras E The bidirectional relationship between sleep problems and internalizing and externalizing problems in children with ADHD: a prospective cohort study. Sleep Med (2016) 17:45 –51. 10.1016/j.sleep.2015.09.019 26847973

[112] Bussalb A Congedo M Barthelemy Q Ojeda D Acquaviva E Delorme R Clinical and experimental factors influencing the efficacy of neurofeedback in ADHD: a meta-analysis. Front Psychiatry (2019) 10:35. 10.3389/fpsyt.2019.00035 30833909 PMC6388544

[113] Riesco-Matias P Yela-Bernabe JR Crego A Sanchez-Zaballos E What do meta-analyses have to say about the efficacy of neurofeedback applied to children with adhd? Review of previous meta-analyses and a new meta-analysis. J Atten Disord (2019) 1087054718821731. 10.1177/1087054718821731 30646779

